# High Aspect Ratio and Light-Sensitive Micropillars Based on a Semiconducting Polymer Optically Regulate Neuronal Growth

**DOI:** 10.1021/acsami.1c03537

**Published:** 2021-05-13

**Authors:** Frano Milos, Gabriele Tullii, Federico Gobbo, Francesco Lodola, Francesco Galeotti, Chiara Verpelli, Dirk Mayer, Vanessa Maybeck, Andreas Offenhäusser, Maria Rosa Antognazza

**Affiliations:** † Institute of Biological Information Processing IBI-3, Forschungszentrum Jülich GmbH, 52425 Jülich, Germany; ‡ RWTH University Aachen, 52062 Aachen, Germany; § Center for Nano Science and Technology@PoliMi, Istituto Italiano di Tecnologia, 20133 Milano, Italy; ∥ Physics Department, Politecnico di Milano, Piazza L. Da Vinci 32, 20133 Milano, Italy; ⊥ Istituto di Scienze e Tecnologie Chimiche G. Natta (SCITEC), Consiglio Nazionale delle Ricerche, 20133 Milano, Italy; # Istituto di Neuroscienze, Consiglio Nazionale delle Ricerche, 20133 Milano, Italy

**Keywords:** conjugated polymers, topography, embryonic cortical neurons, microstructured cell interfaces, tissue engineering, cell optical excitation, cell−substrate interface

## Abstract

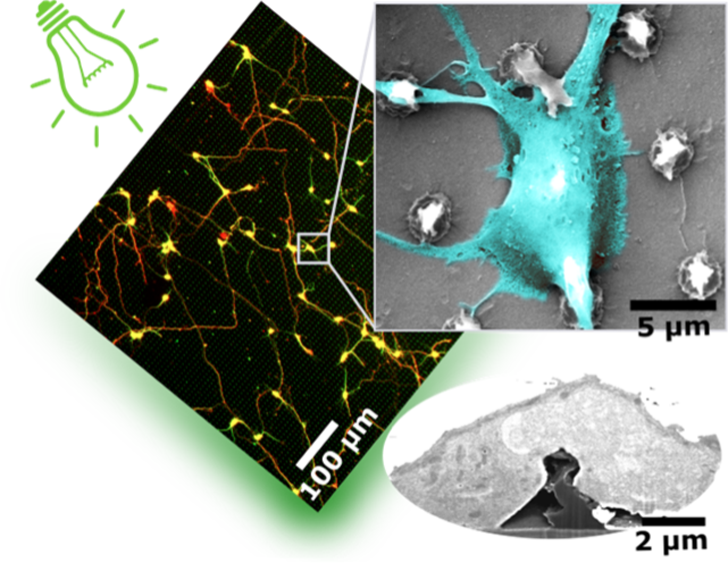

Many nano- and microstructured devices capable of promoting neuronal growth and network formation have been previously investigated. In certain cases, topographical cues have been successfully complemented with external bias, by employing electrically conducting scaffolds. However, the use of optical stimulation with topographical cues was rarely addressed in this context, and the development of light-addressable platforms for modulating and guiding cellular growth and proliferation remains almost completely unexplored. Here, we develop high aspect ratio micropillars based on a prototype semiconducting polymer, regioregular poly(3-hexylthiophene-2,5-diyl) (P3HT), as an optically active, three-dimensional platform for embryonic cortical neurons. P3HT micropillars provide a mechanically compliant environment and allow a close contact with neuronal cells. The combined action of nano/microtopography and visible light excitation leads to effective optical modulation of neuronal growth and orientation. Embryonic neurons cultured on polymer pillars show a clear polarization effect and, upon exposure to optical excitation, a significant increase in both neurite and axon length. The biocompatible, microstructured, and light-sensitive platform developed here opens up the opportunity to optically regulate neuronal growth in a wireless, repeatable, and spatio-temporally controlled manner without genetic modification. This approach may be extended to other cell models, thus uncovering interesting applications of photonic devices in regenerative medicine.

## 1 Introduction

Neuronal cells exist in a complex environment consisting of various mechanical, topographical, electrical, and chemical cues that guide their functions and behavior.^1^ Incorporating these functional features into new “smarter” biointerfaces could tackle the various challenges arising from interfacing living cells with artificial substrates for applications in tissue engineering, regenerative medicine, and bioelectronics.^2−4^ The advancements in the fabrication technology have enabled the creation of highly ordered micro- and nanoscale patterns providing topographical stimulation of neuronal adhesion, differentiation, neurite outgrowth, and guidance *in vitro*.^5^ Current platforms are commonly fabricated using different materials ranging from metals and inorganic semiconductors employed in bioelectronic devices to organic polymers for tissue engineering and regenerative medicine.^6^ Although electrically active materials have been traditionally employed as three-dimensional (3D) electrodes to achieve sensitive recording/stimulation of neuronal activity, many studies have reported the beneficial effects of electrical stimulation on neuronal differentiation and repair.^7−9^ This led to the realization of electroconductive neural scaffolds as potential nerve repair tools.^10^

Organic semiconducting polymers have attracted considerable interest in tissue engineering and bioelectronics due to their intrinsic optoelectrical properties along with chemical and mechanical compatibility with living tissues.^11,12^ These materials are capable of converting light into an electrical current without requiring an external power source, thereby providing a non-invasive photoelectrically active platform for neural engineering. Among other materials, thiophene-based polymers, such as regioregular poly(3-hexylthiophene-2,5-diyl) (P3HT), offer outstanding optoelectronic properties extensively employed in biohybrid devices for optical actuation of neuronal firing.^13,14^ P3HT has been shown to stimulate growth and differentiation of human induced pluripotent stem cells (hiPSC)-derived retinal ganglion cells when illuminated by green laser or visible spectrum light.^15^ Additionally, P3HT light excitation was previously studied in combination with anisotropic or disordered topographical arrays (e.g., fibers and stripes) to stimulate cell growth and differentiation.^16,17^ Recently, a novel P3HT-based biointerface patterned into high aspect ratio (HAR) micropillars has been obtained using a highly repeatable push-coating technique as a novel contribution in the development of optically excitable microstructures.^18^ The proposed P3HT micropillar arrays provided a biocompatible environment for neuronal cultures and HEK-293 cells, with remarkable changes in the cell morphology. Generally, vertical HAR micro- and nanostructures have been employed to facilitate delivery of biomolecules, stem cell differentiation, and as 3D electrodes to record electrical signals with a high signal-to-noise ratio.^19−22^ However, their application in optically active platforms for active photomodulation of neuronal growth has not been attempted.

In this study, we employed the recently developed P3HT HAR micropillar array in a combinatorial approach harnessing both its microtopography and light responsivity to optically stimulate growth of embryonic cortical neurons. A comprehensive investigation of the cell–micropillar interface and actin cytoskeleton confirmed that P3HT micropillars provide a mechanically compliant environment and achieve a close contact with living cells. Early development of embryonic neurons in response to HAR micropillars alone as well as upon visible light stimulation was characterized and compared to non-structured polymer substrates and optically inert controls. Overall, we report a biocompatible, microstructured, and light-sensitive platform for optically driven, non-invasive modulation of embryonic neuronal growth.

## 2 Experimental Section

### 2.1 Fabrication of Polymer-Based Microstructured Arrays

The fabrication of the polydimethylsiloxane (PDMS) mold and P3HT substrates was conducted, as described in a study by Tullii et al.^18^ Commercial glass/ITO slabs were cut into 14 × 14 mm slides, washed consecutively with distilled water, acetone, and isopropanol in an ultrasonic bath (10 min each), and dried with a N_2_ flux. rr-P3HT was dissolved in *o*-dichlorobenzene (20 g/L) and stirred overnight at 50 °C. A drop of the rr-P3HT solution (1 μL) was deposited onto the cleaned glass/ITO surface and pushed using the micropatterned PDMS mold. After a thermal treatment at 90 °C for 2 min, the mold was gently removed, resulting in a 4 × 5 mm rr-P3HT pillar array surrounded by a flat rr-P3HT region deposited on top of glass/ITO substrates.

In order to fabricate micropillars made of photoinert OrmoComp (OC; Microresist Technology GmbH, Germany), new PDMS molds were prepared by replica molding, using P3HT pillar arrays as templates. This step is necessary since, in contrast to P3HT, the printing process using OrmoComp is expected to damage the PDMS after a few depositions by releasing insoluble pillar fragments into the mold. Therefore, using the original micromachined PDMS would be inconvenient. First, 500 nm of parylene was deposited on top of glass/P3HT pillar arrays by vapor deposition polymerization in order to thicken the structure of pillars, strengthen their tips, and facilitate the mold detachment in the replica molding process. The PDMS precursor was mixed with the curing agent (10:1 volume ratio), degassed in vacuum for 30 min, and the mixture was poured onto the parylene-treated glass/P3HT pillar array. After thermal treatment at 65 °C for 4 h, the PDMS layers were gently removed and washed with ethanol. The obtained stamps were used for push-coating a drop of OrmoComp (10 μL) on top of 14 × 14 mm glass slides previously washed in an ultrasonic bath with distilled water, acetone, and isopropanol for 10 min each. Given the UV curable nature of OrmoComp, the samples were treated with an UV lamp (Hamamatsu Lightningcure LC8, 365 nm, 4.5 W/cm^2^) for 10 s by shining the light from the PDMS side. The molds were then removed to obtain a 4 × 5 mm array of OC pillars, surrounded by a flat OC region, deposited on glass substrates.

### 2.2 Electrochemical Impedance Spectroscopy

Neuronal cells were plated on ITO/P3HT pillars and ITO/P3HT flat substrates at a density of 215,000 cells/cm^2^ and cultured for 13 DIV prior to measurements. Optical microscopy images were acquired using an inverted microscope (Nikon Eclipse Ti–S) in order to check that the samples were completely covered by neurons. Control samples without cells were treated with the same protocol employed for the preparation of neuronal cultures but without adding the cell suspension to the growing medium. ITO/P3HT pillars and ITO/P3HT flat samples with and without primary cortical neurons were placed as the working electrode in an electrochemical cell in a three-electrode configuration, comprising a platinum wire as the counter electrode and saturated-KCl Ag/AgCl as the reference electrode. The planar glass/ITO/P3HT part of the flat/pillar devices was removed in order to guarantee that only the impedance contribution from the pillar array is taken into account. EIS measurements were carried out in Krebs–Ringer HEPES extracellular solution [composition (mM): 135 NaCl, 5.4 KCl, 5 HEPES, 10 glucose, 1.8 CaCl_2_, 1 MgCl_2_] at room temperature using an Autolab potentiostat PGstat 302 (Metrohm). Impedance spectra were recorded in the 0.01 Hz to 100 kHz frequency range, with an AC amplitude of 0.02 V, at the electrochemical equilibrium, that is, at DC potential values corresponding to the electrode open-circuit potential (0.01 and 0.12 V for rr-P3HT flat with and without neurons, respectively; 0.07 and −0.01 V for rr-P3HT pillars with and without neuronal cells, respectively). Nova 1.8 software was used for data analysis.

### 2.3 Primary Cell Culture and Photostimulation

Prior to cell seeding, all substrates were sterilized in 70% ethanol and dried with N_2_ gas to ensure aseptic conditions. Sterilized substrates were treated with a non-specific poly-l-lysine (PLL, 1 μg/mL in deionized water) coating overnight at 4 °C and washed twice with deionized water. Primary cortical neurons were isolated from E18 Wistar rat embryos in accordance with the Landesumweltamt fur Natur, Umwelt und Verbraucherschutz, Nordrhein-Westfalen, Recklinghausen, Germany (81-02.04.2018.A190). Briefly, cortical neurons were dissociated by trypsinization followed by mechanical trituration and suspended in Neurobasal medium (Life Technologies) supplemented with 1% (vol/vol) B-27 (Invitrogen), GlutaMAX (0.5 mM, Invitrogen), and gentamycin antibiotic (50 μg/mL). PLL-coated ITO/P3HT substrates were seeded at a density of 150 cells/mm^2^ to distinguish individual cells. Alternatively, a fluorescent filamentous actin (F-actin) marker Lifeact-RFP^23^ was introduced. Approximately 3–5 million cells were resuspended in 100 μL of Nucleofector transfection solution and loaded with 3–6 μg of Lifeact-RFP cDNA plasmid. The suspended cells were transfected using the Amaxa Nucleofector device, program G-013 and seeded onto ITO/P3HT substrates to visualize the actin cytoskeleton.

Photostimulation was applied on DIV1 and 2 on primary neuronal cultures on both flat and pillar P3HT substrates. Standard glass substrates and OC pillar substrates were subjected to the same treatment to serve as photoinert controls. Light stimulation was conducted using the Axio Observer.Z1 (Zeiss) microscope equipped with an incubation chamber (PeCon) with a temperature, CO_2_, and humidity control. The setup was equipped with a Colibri LED light source (Zeiss). Green (555/30 nm) and red (625/30 nm) light-emitting diodes (LEDs) with a photodensity of 0.5 mW/mm^2^ were applied through the medium in 1 s pulses every 1 min for 1 h each day.

### 2.4 Viability Assay

Cell viability was determined using the calcein AM/ethidium homodimer assay. Cells were washed with preheated Neurobasal base medium (without supplements) and incubated 20 min at 37 °C with calcein AM and ethidium homodimer (1 μM in Neurobasal). After incubation, cells were washed twice with warm Neurobasal base medium and imaged using a 10× water immersion objective (N-Achroplan, 0.3 NA, Zeiss) or a 20× water immersion objective (N-Achroplan, 0.5 NA, Zeiss).

### 2.5 Scanning Electron Microscopy

Scanning electron microscopy (SEM) was used to investigate cell–topography interactions on the nanoscale. Cells were washed three times with preheated phosphate-buffered saline (PBS) and fixed with glutaraldehyde (3.2% w/v in preheated PBS) for 15 min at RT. After fixation, samples were thoroughly washed with PBS and deionized water followed by dehydration in increasing concentrations of ethanol: 10, 30, 50% (5 min each), 70, 90, and 95% (15 min each). The samples were then stored in 100% ethanol and prepared using critical point drying (030, BAL-TEC Company). A thin layer of platinum was deposited via sputter deposition (K575X Sputter Coater, Quorum EMITECH) to eliminate charge effects. SEM images were made from the top and with a 45° under beam acceleration of 3–10 kV using SE and inLens detectors (1550VP, Zeiss and Helios 600i NanoLab dual-beam, FEI).

### 2.6 Focused Ion Beam/SEM

Focused ion beam (FIB) in combination with SEM was employed for high-resolution characterization of the cell–substrate interface. In order to preserve the structural integrity of the specimen as well as to enable high-resolution imaging of intracellular structures, samples were stained and prepared using a resin embedding method, as previously described in Belu et al.^24^ After fixation, samples were washed with PBS and cacodylate buffer following treatment with osmium tetroxide (OsO_4_) and uranyl acetate (depleted UrAc, 2% in water) to enhance visualization of cellular structures.^25^ The samples were preserved by resin plastification using a mixture of Epon 812, DDSA, MNA, and DMP-30 solutions and coated with a thin layer of platinum to eliminate charge effects. A complementary dual beam system containing both electron and ion beams (Helios NanoLab dual-beam 600i, FEI) was used for FIB cross-sectioning and visualization of the cell–substrate interface. A region of interest was covered with a platinum layer to eliminate surface charging and to protect cellular structures. A 0.4 μm thick layer of platinum was deposited via electron beam-induced deposition (3 kV, 1.4–11 nA) at 0° fixed stage followed by a 0.4 μm layer deposition at 52° tilt via ion beam-induced deposition (30 kV, 0.23–2.5 nA). A 9.3 nA gallium ion beam at 30 kV was used for cross-section milling, followed by polishing at 30 kV and 0.079 or 0.08 nA. SEM was performed using the electron column at 3 kV with secondary and back-scattered electron detectors.

### 2.7 Fluorescent Immunocytochemistry and Image Analysis

After 3 DIV, the cells were fixed with 4% paraformaldehyde (Sigma-Aldrich) diluted in PBS for 10 min at room temperature (RT) and permeabilized with 0.3% Triton X-100 (Sigma-Aldrich) in blocking buffer (BB, 2% bovine serum albumin and 2% heat-inactivated goat serum diluted in PBS, Sigma-Aldrich) for 15 min at RT. Unspecific binding sites were blocked with BB at RT for 1 h. Afterward, substrates were rinsed and incubated with primary antibodies. Primary antibodies against β-III-tubulin (cortical marker; 2 μg/mL, rabbit-T2200, Sigma-Aldrich) and Tau-1 (axonal marker; 4 μg/mL, mouse-PC1C6, Sigma-Aldrich) were used to visualize the neuronal morphology. The secondary antibodies used were goat anti-rabbit Alexa Fluor 488 (Life Technologies) and goat anti-mouse Alexa Fluor 633 (Life Technologies) both diluted to 4 μg/mL in BB. Fluorescence microscopy was performed using a 10× objective (Plan-Apochromat, 0.45 NA, Zeiss) and analyzed using Fiji’s NeuronJ plugin.^26^ Neurons that formed clusters were not included in the analysis. Neurite directionality and alignment were analyzed using the ImageJ fast Fourier transform (FFT), as previously described.^27^ Briefly, the fluorescent images of β-III-tubulin-positive neurons were processed in ImageJ to yield an FFT image with pixel intensity distribution in the frequency domain. ImageJ’s *Oval Profile* plugin was used to sum the pixel intensities along a circle with its origin in the center of the FFT image (800 pixels radius), and the obtained radial sum intensity was averaged across multiple images. At least 10 micrographs from three independent cultures were analyzed for both P3HT flat films and P3HT pillar substrates.

High-resolution analysis of cell–micropillar interactions was conducted on Lifeact-RFP-expressing cells using a 63× oil immersion objective (plan-apochromat, 1.4 NA, Zeiss) on an Axio Observer LSM 880 equipped with an Airyscan detector. Alternatively, point contact adhesions were visualized using the anti-paxillin antibody [Y113]. Cells were additionally stained with TRITC-phalloidin to visualize the actin cytoskeleton. Images were acquired with a 63× oil immersion objective (plan-apochromat, 1.4 NA, Zeiss) on a confocal laser-scanning microscope.

### 2.8 Statistical Analysis

Data were analyzed using R software. Quantitative measurements were evaluated via a Shapiro–Wilk test to assess normality and then compared using the nonparametric Mann–Whitney *U*-test or the parametric Student’s *t*-test. Multiple comparison correction was performed using the Holm–Bonferroni method. All boxplots are of Tukey type with the median denoted as a line and the mean as a black cross. A *p*-value less than 0.05 was considered statistically significant.

## 3 Results and Discussion

### 3.1 Characterization of HAR Micropillars

**Figure 1:**
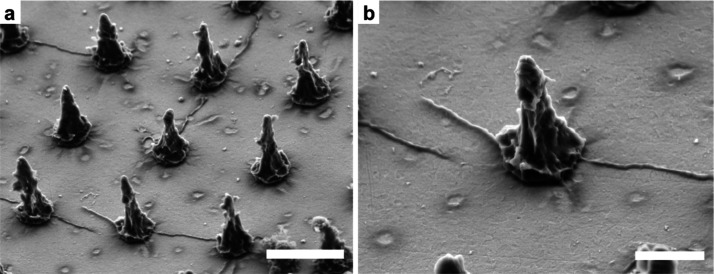
P3HT micropillar array. Representative SEM images of a micropillar array (a) and an individual pillar (b). Micropillars had a conical shape with a high degree of nanoscale roughness on their sidewalls. Images were acquired with a 45° tilt angle. Scale bars: (a) 5 and (b) 2 μm.

P3HT micropillar arrays were fabricated using the push-coating technique, as previously reported.^18^ The push-coating method takes advantage of a PDMS stamp patterned with the negative pillar morphology (*i.e.*, microholes) using femtolaser micromachining to finely tune the structural parameters of the polymer pillars (size, aspect ratio, 3D shape, and pitch) as these are known to strongly affect cell adhesion, viability, and proliferation.^21,28^ This method is suitable for the fabrication of both planar films^29^ and nano/microstructures.^18,30^ By taking advantage of the excellent adaptability of the push-coating to different polymers, we extended this approach to fabricating micropillar arrays made of a hybrid organic/ceramic polymer, OrmoComp. OrmoComp is a commercial polymer with proven biocompatibility^31,32^ that lacks optoelectronic properties. The push-coating technique was employed to fabricate micropillars with identical geometrical characteristics to those on P3HT arrays. For this purpose, the PDMS mold was reproduced by replica molding and used for stamping the OrmoComp resin on top of glass substrates. OrmoComp micropillar arrays were employed as an optically inert control to decouple the effects of light stimulation from those exerted by topographical cues. Microscale structures made of either polymer (P3HT or OrmoComp) had a conical-like shape with an average pitch (center-to-center distance) of 7.2 ± 0.2 μm (Figures 1a and S1a). The array pitch and the corresponding pillar density (2 pillars/100 μm^2^) were inspired by studies demonstrating that similar pillar density promotes cell spreading without affecting their viability.^33,34^ Conversely, higher density pillars lead to limited cellular adhesion and viability, likely due to a reduced cell–substrate contact.^21,35^ Average pillar height, base diameter, and half-height width were 6.4 ± 0.3, 2.3 ± 0.1, and 1.2 ± 0.2 μm, respectively (Figures 1b and S1b). Moreover, micropillars presented a high degree of nanoscale roughness on their sidewalls determined by the laser process used to dig the microcavities in the PDMS mold.

### 3.2 Neuronal Adhesion on Micropillar Arrays

**Figure 2:**
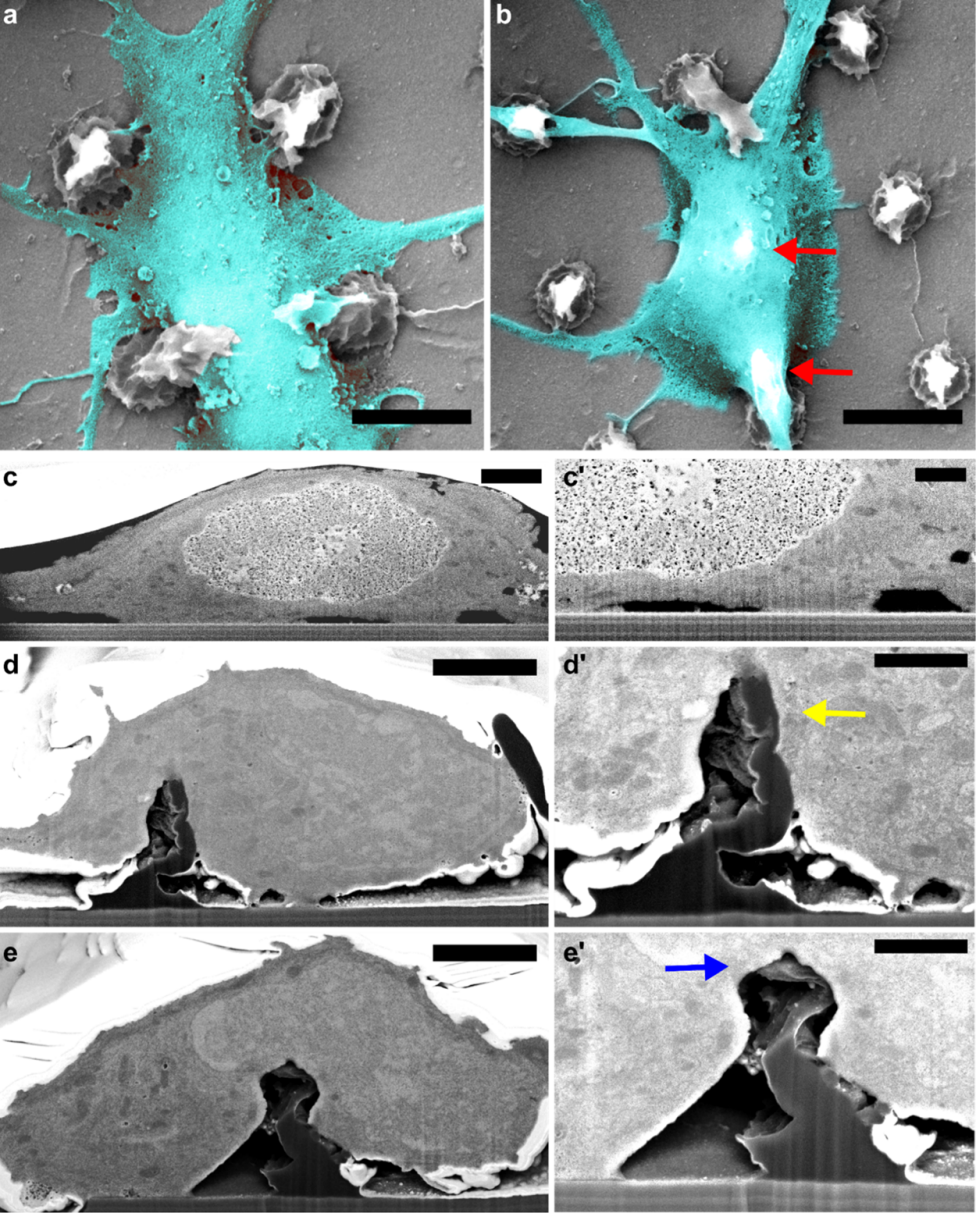
FIB/SEM characterization of the cell–micropillar adhesion. (a) Neuronal soma positioned between the pillars and (b) suspended over two pillars (red arrows). Scale bars: 5 μm. (c,c′) FIB/SEM cross-sections of the neuronal soma positioned on the flat surface. (d,e′) FIB/SEM cross-sections of the neuronal soma positioned on a micropillar at the soma periphery (d,d′) and at the soma center (e,e′). Arrows in (d′,e′) indicate the pillar being pulled toward the center (yellow arrow) and pushed down (blue arrow), respectively. Scale bars: (c,d,e) 2 and (c′,d′,e′) 1 μm.

Vertical HAR structures have been successfully employed for modulating various cell responses, including membrane penetration,^36^ adhesion,^37^ and axon development.^38^ However, HAR structures were also shown to impair cell viability by penetrating cell bodies or hindering their motility.^39,40^ Therefore, embryonic cortical neurons were seeded onto PLL-coated P3HT substrates, and neuronal viability was assessed using the calcein AM/ethidium homodimer assay after 3 DIV (Figure S2a,b). Neither flat nor microstructured P3HT substrates impaired cell viability compared to standard glass controls (Figure S2c), in accordance with previous studies demonstrating the outstanding biocompatibility of P3HT with various cell types both *in vitro* and *in vivo*.^18,41−43^ The neuronal morphology on P3HT micropillars was qualitatively investigated using SEM. Neuronal somas were localized between the pillars (Figure 2a) or partially suspended over the micropillars with apparent membrane spreading in the proximity of the pillar tips (Figure 2b, red arrows), as previously observed on various vertical micro- and nanostructures.^24,36,40^ HAR micropillars seemed to be fully embedded in the cell, while the rest of the membrane reached the flat surface between the pillars. Furthermore, neuronal processes tended to wrap around the pillars (Figure S3a). This resulted in a network of neuritic bundles and numerous branched processes that were anchored to the nanoscale grooves and ridges on pillar sidewalls. Nanoroughness on pillar sidewalls was previously shown to promote formation of 3D neuronal networks and enhance neurite adhesion.^44^

The nature of the cell–micropillar interface was investigated using FIB cross-sectioning to understand how the cell membrane interacts with the micropillar surface. Previous studies have shown that vertical structures on the substrate surface induce membrane wrapping, generate local curvatures of the membrane,^36,45^ and facilitate adhesion.^46^ After 3–5 DIV, neurons were fixed and stained with osmium tetroxide and uranyl acetate to enable visualization of cellular compartments. Cellular structures were preserved in an ultrathin layer of plastic resin^24^ and cross-sectioned via FIB. When the soma was positioned between the pillars, the cell membrane attached at multiple sites to the substrate with the rest of the membrane being suspended above the surface (Figure 2c,c′). Somas that were suspended on the pillars substantially deformed the more flexible upper part of the micropillars, whereas the base of the pillar remained unchanged. Moreover, we observed that neurons often pulled and bent the micropillars (Figure S3b), as previously observed on HAR structures.^47^ Although vertical HAR structures are often broken by cellular forces,^48^ the conical shape of the micropillars presented here likely ensures both stability due to the microscale pillar base as well as reduced stiffness of the nanoscale pillar tip. No membrane rupturing was observed, and the organelles were readily present in the vicinity of micropillars, in accordance with previous studies on similar structures.^49^ Furthermore, we observed that the manner of pillar deformation was dependent on the relative position of the pillar with respect to the soma. When the pillar was positioned near the soma periphery, it was pulled toward the center (Figure 2d,d′, yellow arrow), whereas the pillars positioned in the center of the soma were pushed down (Figure 2e,e′, blue arrow). The pulling at the periphery is likely mediated by cytoskeletal forces dragging the junctional membrane (that facing the substrate) toward the center,^50^ while the relatively stiff nucleus could push the pillar down.^51^ In fact, even though the upper part of the pillars was easily deformed by the cell membrane, the larger pillar base remained unperturbed and stable, which induced upward bending of the nuclear membrane (Figure 2e). Since cellular traction forces are mainly exerted on the pillars’ upper part (Supporting Information), the bending stiffness was roughly estimated by approximating the upper half of a micropillar to a rodlike structure.^52^ The calculated bending stiffness of the upper part was in the range of 1.1–33.9 μN/μm, similar to parylene C micropillars (4.8–28 μN/μm) previously used for quantification of cell forces^53^ and substantially smaller than the values obtained for inorganic materials, like silicon nanowires bundles (100–1200 μN/μm).^54^

Thus, P3HT micropillars provide a complex interface consisting of a compliant upper part easily deformed by cytoskeletal forces and a stable base, capable of inducing substantial membrane rearrangements around the pillars. Moreover, Tullii et al.^18^ measured a higher membrane capacitance on P3HT micropillars, which might be related to membrane wrapping around the micropillars leading to a higher junctional membrane/cell volume ratio.^55^ We quantified the membrane wrapping induced by P3HT micropillars as the ratio between the total junctional membrane length and the cell diameter, where a higher ratio denotes more membrane bending. For this purpose, the membrane facing the substrate was defined as the junctional membrane, while the rest of the membrane was disregarded since it was not affected by the microstructures (Figure S3c). The ratio between the total junctional membrane and the cell diameter was 1.12 ± 0.02 and 1.85 ± 0.13 for somas on the flat surface and pillars, respectively, indicating that the pillars induced significantly more pronounced membrane bending compared to the flat surface (*p* = 0.005; Figure S3d). Thus, increased membrane bending along with a smaller cell diameter (8.91 ± 0.28 μm on pillar compared to 17.8 ± 1.43 μm on flat) indicates a higher membrane/volume ratio. In fact, approximating the cell to a partial hemisphere and taking into account its height and radius, the calculated junctional membrane/volume ratio of cells on pillars is 0.09 ± 0.01 μm^–2^, four times higher than that for cells on the flat surface (0.02 ± 0.003 μm^–2^). This result is in accordance with a sizeable stretching of the soma area on identical micropillars observed in a previous study.^18^

### 3.3 Quantitative Evaluation of Neuronal Morphology

**Figure 3:**
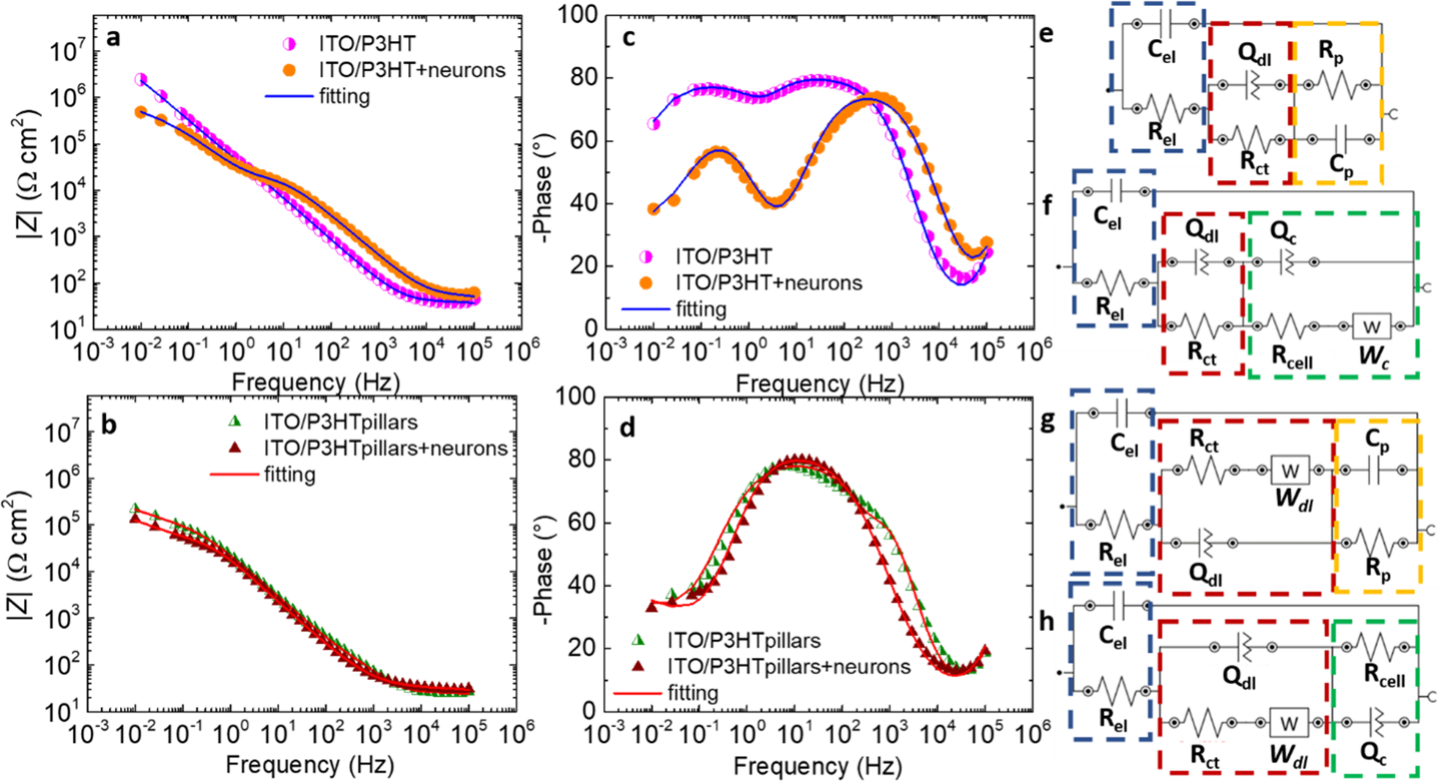
EIS characterization. Impedance modulus *|Z|* and phase angle recorded during EIS experiments for ITO/P3HT (a,c) and ITO/P3HT pillars (b,d) with and without cortical neurons. *|Z|* values were normalized to the geometrical and to the estimated effective area of the sample, in the flat and pillar cases, respectively. Equivalent circuits employed to model the experimental data related to the ITO/P3HT (e), ITO/P3HT + neurons (f), ITO/P3HT pillars (g), and ITO/P3HT pillars + neurons (h) systems. Numerical values of the circuital components are reported in Table S1. χ^2^ values: 0.08 and 0.05 for ITO/P3HT samples, with and without cells, respectively; 0.06 for cell-covered and uncovered ITO/P3HT pillars.

In order to corroborate and quantify neuronal morphological changes observed in the SEM study, electrochemical impedance spectroscopy (EIS) measurements were performed. EIS has been often employed as a quantitative analytical method to investigate the cell morphology, growth, and adhesion on electrodes, as well as differentiation, cell–cell and cell–matrix interactions, and cell motility.^55−62^ EIS measurements were performed on P3HT-based substrates displaying a high cell coverage to minimize the impedance contribution of the electrode/electrolyte interface (Figure S4). Figure 3a–d shows the representative Bode plots obtained for ITO/P3HT and ITO/P3HT pillars with primary cortical neurons compared to substrates without neurons. Neurons seeded on flat P3HT electrodes induced a decrease of *|Z*| in the low-frequency region (<1 Hz) and an increment of *|Z|* between 10 and 10^4^ Hz (Figure 3a). We can consider only the latter *|Z|* variation as the contribution of the cells to the electrode impedance since at frequencies <10 Hz, the *|Z|* spectrum is dominated by the electrode, as previously observed on platinum and gold electrodes.^61,63,64^ Interestingly, in the case of P3HT pillars devices, *|Z|* values with and without cells in the mid-range frequency region were comparable (Figure 3b). The *|Z|* spectra of P3HT pillar devices were normalized to their effective area by approximating the pillars as perfect cones, and it is about 35% higher with respect to the sample geometrical area. The impedance phase angle diagram of the planar P3HT devices has two maxima at intermediate (10 ÷ 10^3^ Hz) and low (∼10^–1^ Hz) frequencies, two minima around 1 and 10^4^ Hz, and displays a decrease of about 20–30° at frequencies <10^3^ in neuron-covered devices (Figure 3c). The microstructured P3HT electrode, both with and without cells, shows two minima at low (∼10^–1^ Hz) and high (>10 kHz) frequencies, a maximum around 10 Hz, and a maximum at 10^3^ Hz that disappears after cell plating (Figure 3d). In order to model the behavior of the electrode/electrolyte/cell interface, an equivalent circuit fitting of the Bode plots was performed (Figure 3e–h), obtaining very good agreement with the experimental data (Figure 3a–d). The equivalent circuits associated with different conditions are composed of four different circuital loops (Figure 3e–h): (i) capacitance (*C*_el_) and resistance values (*R*_el_) associated with the electrolyte activity (Figure 3e–h, dashed blue regions); (ii) the Helmholtz double-layer activity loop, given by a combination of a constant phase element (CPE, *Q*_dl_) and charge-transfer resistance (*R*_ct_, P3HT flat without and with cells; Figure 3e,f, dashed red regions) or by a combination of *Q*_dl_, *R*_ct_, and a Warburg element (*W*_dl_, P3HT pillars without and with cells; Figure 3g,h, dashed red regions); (iii) *R*_p_ and *C*_p_ values that could be associated with the presence of surface states mediating the charge transfer to the solution, without cells (Figure 3e,g, for P3HT flat and pillars, respectively, dashed yellow regions);^65^ and (iv) the equivalent circuit loop that describes the cells, comprising a CPE (*Q*_c_)-resistor (*R*_cell_) (P3HT pillars; Figure 3h, dashed green region) or a CPE (*Q*_c_)-resistor (*R*_cell_) with a Warburg element (*W*_c_) connected in parallel (flat P3HT; Figure 3f, dashed green region). CPE was employed both to take into account the non-ideal behavior of the polymer/electrolyte interface double-layer capacitance,^66^ as well as to describe the impedance of living cells, in agreement with the existing literature.^63^ The introduction of a Warburg element, instead, reflects the presence of different charge-transfer dynamics, ascribed to a different arrangement of cells on the electrode topography.^67^ Numerical values obtained by the fitting are reported in Table S1.

**Figure 4:**
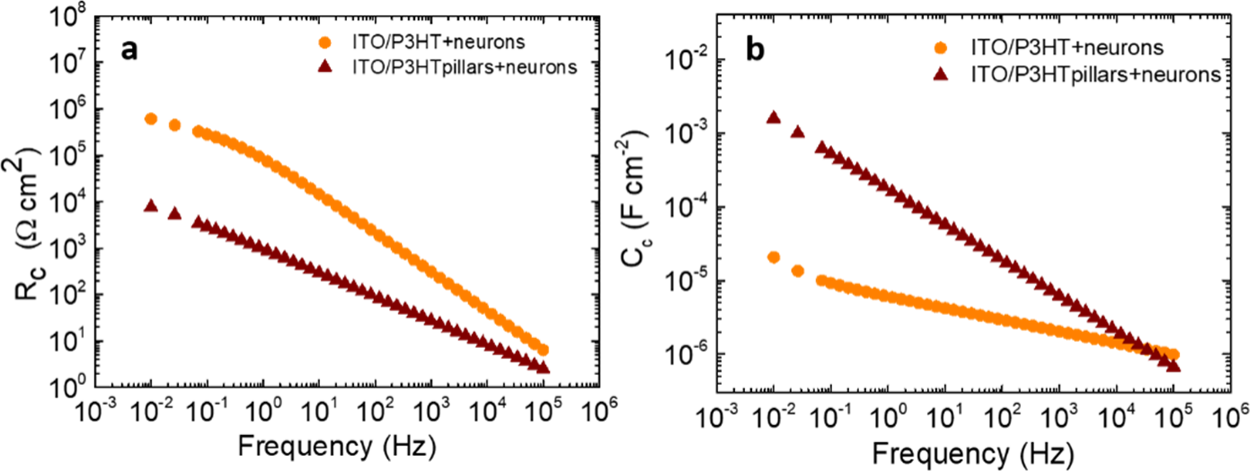
Cell resistance and capacitance. Resistance (*R*_c_) (a) and capacitance (*C*_c_) (b) values, obtained by the combination of circuital parameters (Table S1) of the cell equivalent circuital loops depicted in Figure 3, as a function of the frequency. *R*_c_ and *C*_c_ values were normalized to the geometrical and to the estimated effective area of the sample, in the flat and pillar cases, respectively.

In order to obtain a deeper understanding about the distinct morphology and adhesion of cortical neurons on the electrode topographies, the capacitance and the resistance values of the cell circuital loops (*C*_c_ and *R*_c_, respectively) were calculated by combining the different circuital terms according to the following expressions
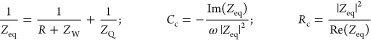
where *Z*_eq_, *Z*_Q_, and *Z*_W_ denote the total impedance, the CPE, and Warburg impedance values, respectively. *R*_c_ and *C*_c_, evaluated from the cell circuital loops reported in Figure 3f,h, (dashed green regions), are depicted in Figure 4a,b, respectively, as a function of frequency. The two parameters must be evaluated between 10 and 10^4^ Hz, where the cell impedance contribution is predominant (Figure 3a).^61,64^ In this frequency region, *C*_c_ reflects the cell membrane capacitance,^55,61^ and *R*_c_ describes the resistance to the current flowing through the cell/substrate space and intercellular gaps.^60,61^ In the 10 ÷ 10^4^ Hz range, the *R*_c_ value is lower in the micropillar case compared to the flat one, by about 2 order of magnitude at 10^2^ Hz (82 and 1.8 × 10^3^ Ω cm^2^ for the pillars and flat cases, respectively; Figure 4a). A decrease in the *R*_c_ parameter can be associated to an increase in both the cell–substrate distance and the intercellular gap, as observed on different cellular models,^58−60^ including neurons derived from neural progenitor cells,^55^ and is consistent with a prevalence of neurons suspended over the P3HT microstructures. In the same frequency region, the *C*_c_ parameter is always higher when the neurons are plated on the P3HT micropillars (1.7 × 10^–5^ and 2.8 × 10^–6^ F cm^–2^ at 10^2^ Hz for the pillars and flat, respectively, Figure 4b). *C*_c_ was found to be directly proportional to the cell membrane to cell volume ratio.^55,57^ Therefore, higher *C*_c_ obtained in the P3HT micropillar case can be reasonably ascribed to a higher junctional membrane/volume ratio, confirming the results derived from SEM analysis.

### 3.4 Actin Cytoskeleton and Adhesions

**Figure 5:**
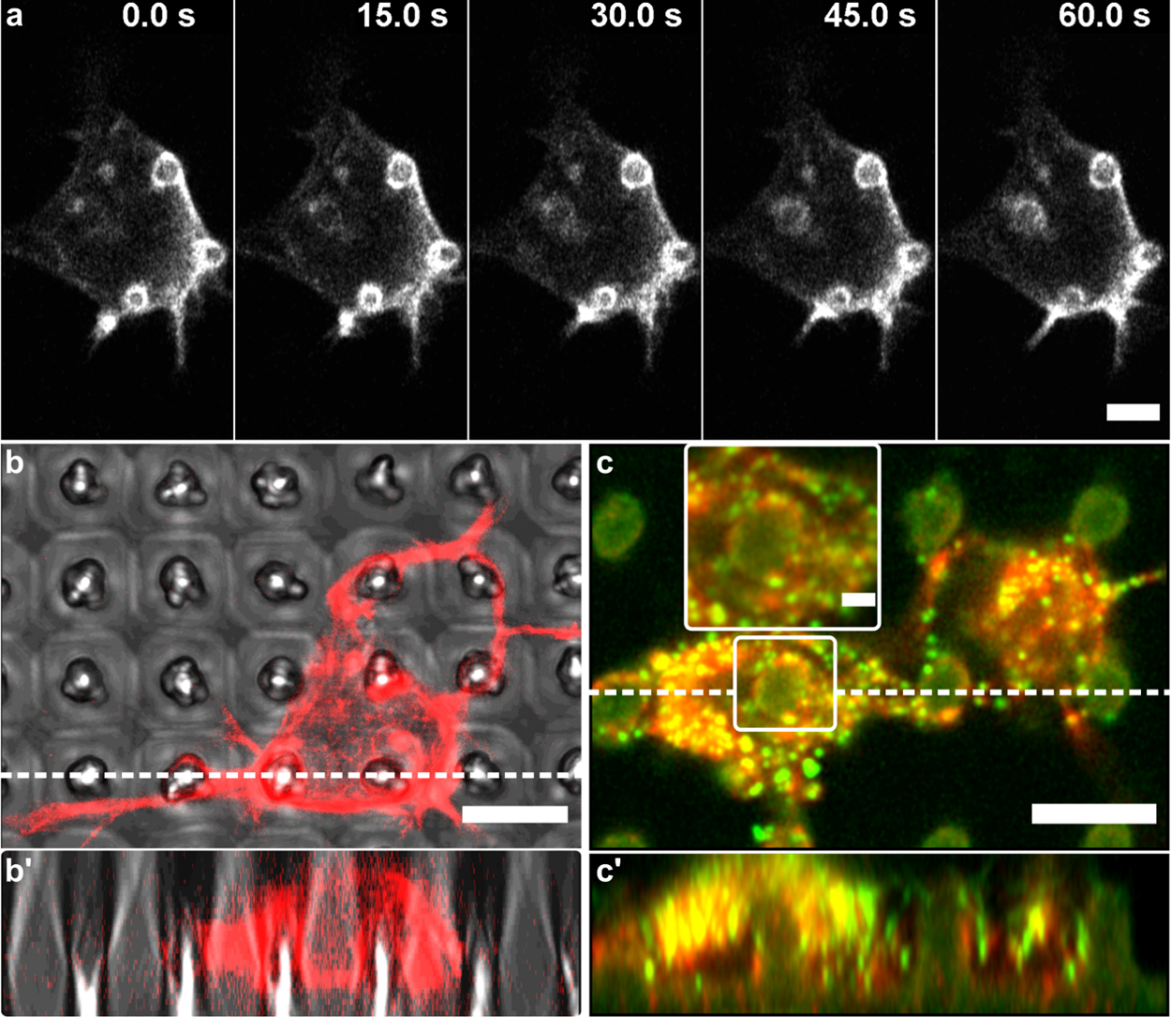
Actin rings and paxillin adhesions on P3HT micropillars. (a) Time-lapse sequence of stable F-actin structures around the pillars. Additionally, the formation of a fourth ring can be observed. (b,b′) F-actin ring-like accumulations formed around the micropillars indicate membrane wrapping. (c,c′) These structures often overlapped with paxillin-rich adhesions (zoomed-in inset; green puncta). Images in (b′,c′) are Z-stack orthogonal projections of 30 slices (400 and 200 nm thickness, respectively), along the dashed lines in images (b,c). Scale bars: (a–c′) 5 μm; inset 2 μm. Cells in (a,b) were transfected with a fluorescent F-actin marker (Lifeact-RFP). Cells in image (c) were stained with TRITC-phalloidin (actin, red) and anti-paxillin antibodies (green).

The analysis of the cell–micropillar interface was further focused on the intracellular architecture that might promote and stabilize membrane rearrangements around the pillars. Primary cortical neurons were transfected with a fluorescent F-actin marker (Lifeact-RFP) and imaged using high-resolution confocal microscopy. Actin cytoskeleton is involved in many cellular behaviors and was shown to be significantly affected by surface topography.^68,69^ We observed F-actin accumulations in the soma visible as rings around the micropillars (Figure 5a). Similar structures were observed around 3D nanostructures^32,70^ and were associated with enabling pillar engulfment,^50^ thereby mediating the mechanical contact of the membrane to the pillar.^71,72^ Moreover, F-actin accumulations around nanopillars above 400 nm in diameter were also associated with the increased membrane area at nanopillar locations, while on smaller nanopillars, F-actin accumulations were associated with high membrane curvature.^45^ Time-lapse imaging of Lifeact-RFP-expressing neurons showed that these structures are relatively stable due to the increased membrane area since curvature-dependent F-actin accumulations are highly dynamic structures (Figure 5a).^45^ Moreover, confocal imaging of Z-stack slices showed that F-actin distributes along the pillar sidewalls following the pillar shape closely (Figure 5b,b′). Finally, paxillin-rich adhesions were often overlapped with encircling F-actin accumulations (Figure 5c, inset). These were present both in the soma and in the neurites and were mostly localized on pillar sidewalls (Figure 5c′), indicating strong adhesion to the pillars. Moreover, there appeared to be substantially more paxillin puncta in the soma positioned on the pillar (Figure 5c, left) compared to the one positioned between the pillars (Figure 5c, right), further indicating the importance of both the actin cytoskeleton and paxillin adhesions in mediating the mechanical coupling of the membrane to the pillar. Thus, neuronal cells interact strongly with mechanically compliant P3HT micropillars often deforming them to achieve a close contact. The following sections explore the potentials of the presented interface by exploiting both its microstructured topography as well as the optoelectronic properties of the photoactive P3HT polymer.

### 3.5 Neuronal Development on P3HT Arrays

**Figure 6:**
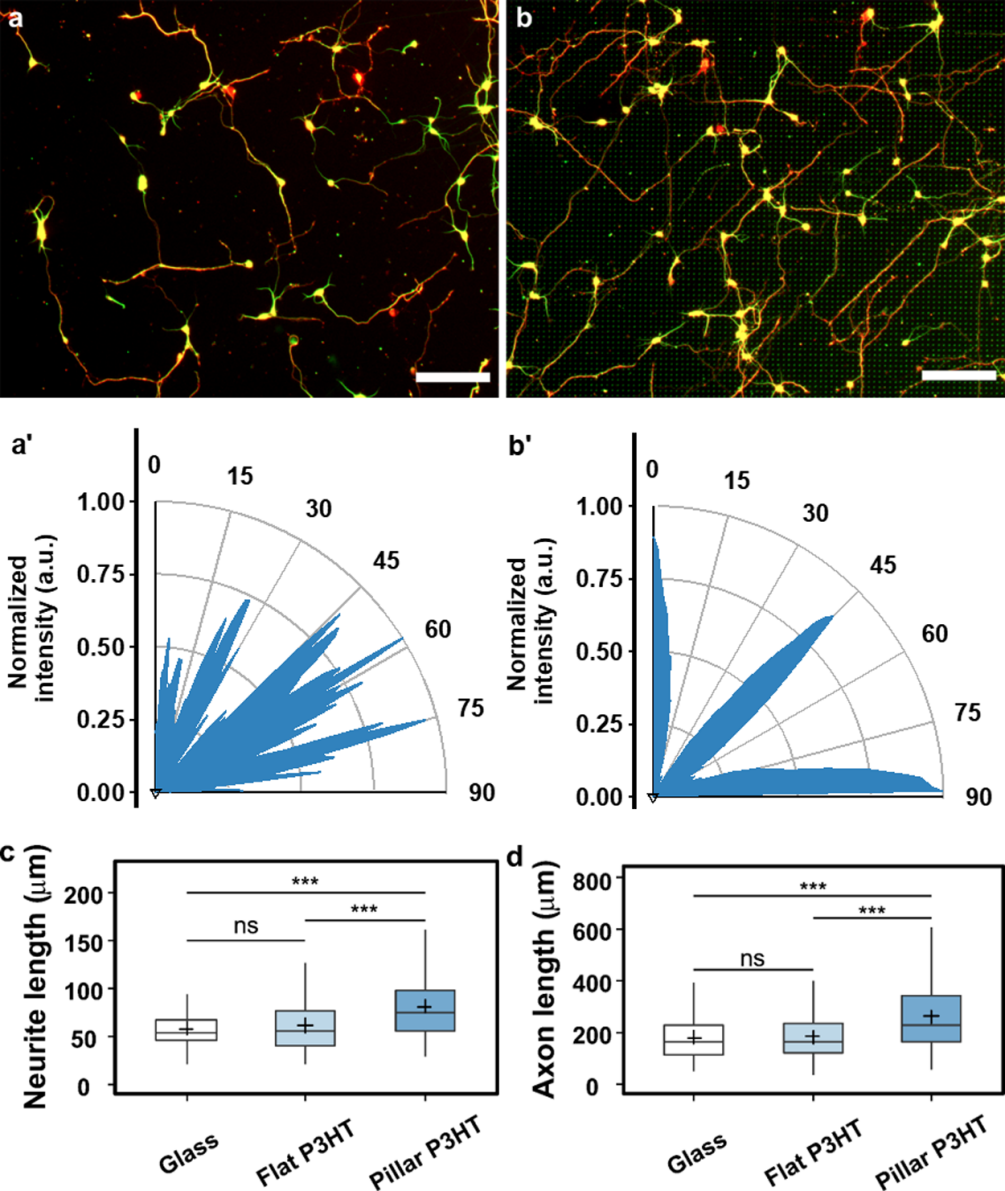
Neuronal growth on P3HT micropillars. Cortical neurons cultured on (a) flat P3HT and (b) P3HT micropillars after 3 DIV. Neurons were fixed and fluorescently labeled for β-III-tubulin (green) and Tau-1 (red). Scale bar: 100 μm. Lower panels (a′,b′) represent the FFT-generated angle distribution of neurite alignment on flat and micropillar arrays, respectively. (c) Average neurite length. (d) Axon length. Number of neurons analyzed: glass = 257, flat P3HT = 225, and pillar P3HT = 229. Data were compared using the nonparametric Mann–Whitney *U*-test with Bonferroni–Holm multiple comparison correction (0.05 significance level). ***p < 0.001, ns—not significant.

Neuronal development on a highly ordered P3HT micropillar topography was investigated and compared to both P3HT flat films and standard glass substrates. Neurons were fixed and stained with anti-β-III-tubulin (cortical marker) and anti-Tau-1 antibodies (axonal marker) after 3 DIV. Cortical neurons showed normal development on both flat and micropillar P3HT substrates with defined axons and dendrites strongly aligned to the underlying micropillar topography (Figure 6a,b). Substrates with defined topographic cues affect cell functions and behaviors in various ways depending on the overall topographical pattern (isotropic or anisotropic) and the geometrical characteristics of the topographical features.^73,74^ For example, discontinuous microtopographies comprising elliptically cross-sectioned microcones have been shown to promote cell and neurite orientation along their elliptical shape.^75^ Therefore, we evaluated neurite alignment using FFT analysis of the images with β-III-tubulin-stained neurons to obtain angular pixel distributions (Figure 6a′,b′). Angular distribution of the pixel intensity clearly shows that neurites were strongly aligned to the topographically dictated angles (*i.e.*, 0 and 90° relative to the direction of the pattern), while those on flat substrates without any topographical cues had a random distribution. Interestingly, although most neurites were aligned at 0/90° relative to the direction of the pattern, we observed a strong peak between 35 and 55°, likely a consequence of the large pitch (∼7 μm), which enables neurites extending between the pillars to bend and/or wrap around the pillars (Figure S3a).^76^ Additionally, the observed diagonal growth can also ensue upon occasional neurite branching upon encountering a pillar.^77^ Time-lapse imaging of Lifeact-RFP-expressing growth cones showed that despite occasional changes in the direction, growth cones generally extended from pillar to pillar (Figure S5a). Thus, micropillar arrays angularly confined neurite outgrowth and might provide regular adhesion points to enable neurite growth from one pillar to the next. This is also in accordance with the observed paxillin adhesions localized on pillar sidewalls (Figures 5c and S5b).

Neurite growth was further quantified by measuring the average neurite length per cell and the axon length. Neurite length was significantly increased by the microtopography, in line with numerous studies of topography-induced responses during neuronal development.^32,78,79^ Moreover, a high level of roughness provided by nanoscale grooves and ridges on P3HT pillar sidewalls could further promote neurite growth^44^ through stronger growth cone coupling that generates traction forces necessary for neurite extension. After 3 DIV, the average neurite length on P3HT micropillars was 84.2 ± 2.85 μm compared to 61.7 ± 1.95 μm on flat P3HT substrates, and to 57.3 ± 1.31 μm on glass controls (Figure 6c). Moreover, cortical neurons on P3HT micropillars had an average axon length of 262.5 ± 9.03 μm, significantly longer compared to 181.3 ± 5.86 and 179.8 ± 5.58 μm on flat P3HT and glass substrates, respectively (Figure 6d). Therefore, P3HT micropillars induce a ∼40% increase in overall neurite growth, further confirming the benefits of ordered microscale topographies on polymeric substrates for promoting neuronal growth.

### 3.6 Photostimulation of Neuronal Development on P3HT Substrates

**Figure 7:**
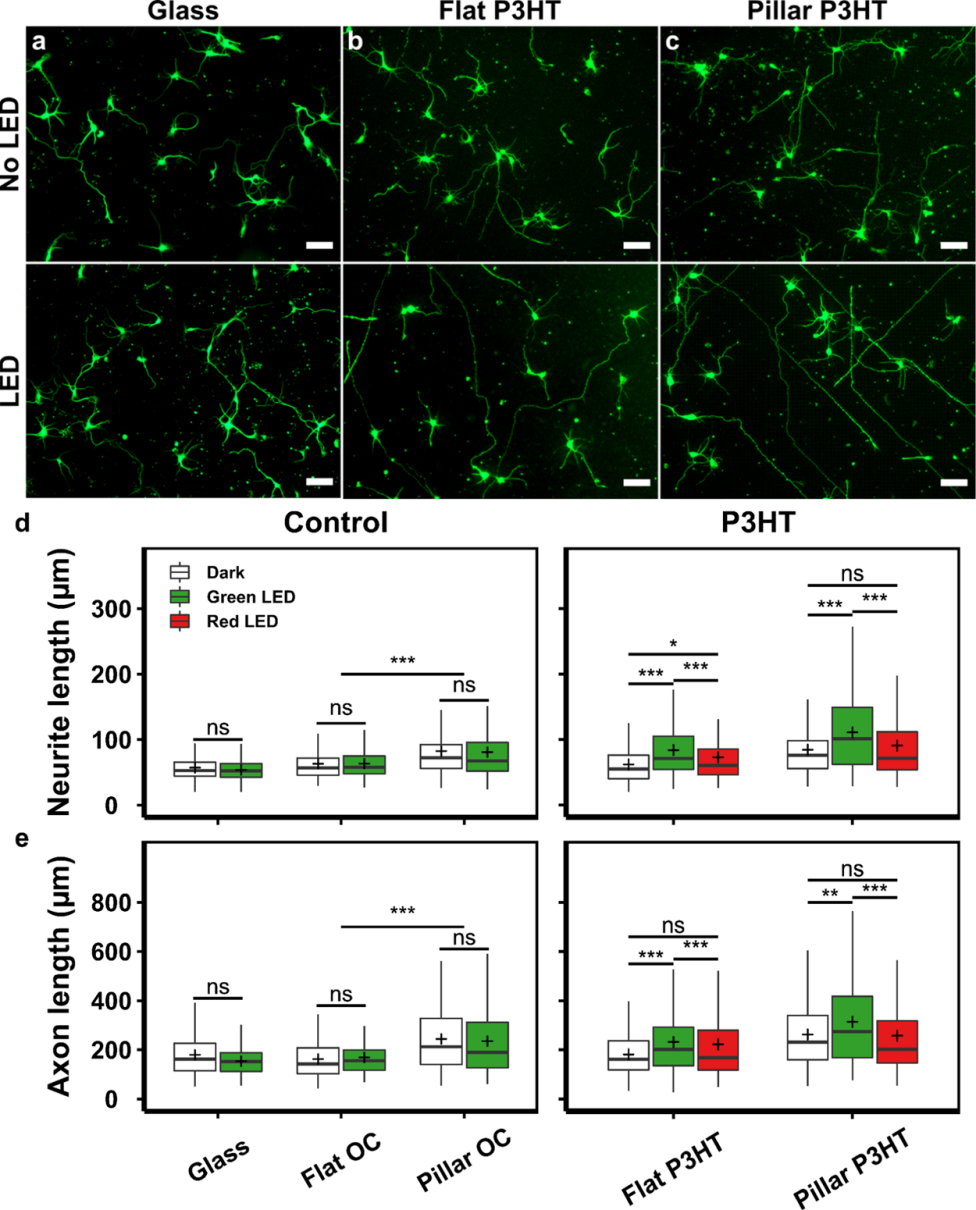
Photostimulation of neuronal growth on P3HT substrates. Representative images of primary neurons (DIV 3) grown with/without photostimulation on (a) glass, (b) flat P3HT, and (c) P3HT micropillars. Neurons were fixed and fluorescently labeled for: β-III-tubulin (green). Scale bar: 50 μm. (d) Average neurite length. (e) Axon length. More than 200 neurons from three independent experiments were analyzed for each substrate and condition. Data in (d,e) were compared using the non-parametric Mann–Whitney *U*-test with Bonferroni–Holm multiple comparison correction (0.05 significance level). *p < 0.05, **p < 0.01, ***p < 0.001, ns—not significant.

The benefits provided by biologically compliant, polymer micropillar topography may be further enhanced by capitalizing on peculiar P3HT optical properties, in the attempt to achieve wireless photostimulation of neuronal growth. Primary embryonic neurons on both flat and pillar ITO-P3HT substrates were stimulated on DIV1 and 2 using a Colibri LED light source (Zeiss) mounted onto an incubated microscope stage. Since P3HT optical absorption spectrum falls in the visible range (450–630 nm) and exhibits a maximum absorption peak at about 530 nm, green (emission peak at 555 nm, with emission profile closely matching P3HT absorption) and red (emission peak at 625 nm, with negligible overlap with P3HT absorption) LEDs were chosen (Figure S6a). Photostimulation (excitation density, 0.5 mW/mm^2^) was applied as 1 s pulses every 1 min for 1 h each day (*i.e.*, on DIV 1 and 2) to limit phototoxicity effects that could impair cell viability. Glass substrates and optically inert OrmoComp micropillar substrates served as controls and were subjected to the same stimulation treatment. No detrimental effects on cell viability were observed as a result of the described stimulation regime (Figure S6b). After 3 DIV neurons showed normal development with clearly defined neurites on all substrates. Neurons grown on glass substrates were relatively unchanged by the photostimulation regime, whereas neurons on photostimulated P3HT substrates appeared to have longer neurites compared to unstimulated cultures (Figure 7a–c). Average neurite length and axon length were statistically analyzed, finding that the photostimulation of neurons growing on control substrates (glass and OrmoComp) had no observable effect on neurite growth (Figure 7d,e). In contrast, average neurite length of neurons growing on P3HT substrates stimulated using a green LED (555 nm) was significantly increased compared to the unstimulated ones. The average neurite length of the stimulated neurons was 83.6 ± 2.33 μm (flat P3HT) and 115.5 ± 4.27 μm (pillar P3HT) compared to 61.7 ± 1.95 μm (flat P3HT) and 84.2 ± 2.85 μm (pillar P3HT) in unstimulated cultures. Similarly, stimulated neurons had significantly longer axons (231.7 ± 6.9 and 313.9 ± 10.4 μm on flat and pillar P3HT, respectively) than the unstimulated ones (181.3 ± 5.9 and 262.4 ± 9.1 μm on flat and pillar P3HT, respectively). Photostimulation using a red LED (625 nm) did not yield significant differences in neurite growth, due to significantly weaker light absorption in this range (Figure S6a). Moreover, since photostimulation did not influence neuronal growth on photoinert substrates (glass and OrmoComp), the observed effects on P3HT substrates can be attributed to the excitation of the active material itself. Additionally, we observed a slight reduction in the number of primary neurites both on illuminated polymer flat substrates and on micropillar arrays, either illuminated or not (Figure S7). Neurite growth and initiation are energetically costly processes involving cytoskeletal rearrangements^80^ and intracellular transport.^81,82^ Thus, the observed reduction in neurite number in polymer samples exposed to light might be related to the high energetic cost of sustaining a large number of longer processes *in vitro*. In addition, it was also suggested that topography itself may play a role^5,83^ since neurite initiation requires a certain amount of physical space to achieve a proper orientation of cytoskeletal filaments to sprout a new process. Thus, the observed decrease in the number of neurites on pillars in dark conditions or exposed to red light (Figure S7) might be related to soma confinement between adjacent pillars acting as obstacles to neurite initiation,^84^ further confirming the synergistic effect of P3HT topography and optical illumination on neuronal growth. Importantly, however, the changes in the neuronal morphology (e.g., decrease in the number of neurites) exerted by HAR pillars did not impair the calcium activity and action potential firing of mature neurons (DIV14), as shown by the patch clamp and intracellular Ca^2+^ measurements performed on neurons on P3HT pillars without optical stimulation (Figure S8). Thus, the presented pillar microtopography does not cause any detrimental alterations in physiological functionality of mature neuronal networks.

Our results unequivocally demonstrate that the microstructured topography of the polymer substrate and its optical responsivity to visible light cooperate to significantly promote neurite outgrowth of embryonic cortical neurons. Although substrate topography and optical stimulation were previously studied separately or in combination with anisotropic or disordered topographical arrays,^16−18^ the presented study originally combines the unique advantages of ordered HAR micropillar arrays, which provide a conformal interface to cell attachment, with P3HT light excitation. A detailed description of the physical–chemical mechanisms active at the photoexcited microstructured interface, as well as of the light-triggered biological pathways leading to the observed enhancement of cellular growth is not straightforward. Previous studies demonstrated that the presence of local positive charges on the cell-culturing surface is beneficial for neurite outgrowth,^85−87^ and even for repair of injured nerves.^88^ Although capacitive coupling may play some role in the phototransduction effect, we can reasonably disregard it as the main mechanism acting on neuronal growth, based on the following considerations: (i) we previously demonstrated that upon photoexcitation with pulses longer than 500 ms, P3HT-based biohybrid interfaces display a photocathodic behavior, thus implying that negative charges preferentially accumulate at the polymer surface and sustain photoactivated electron-transfer reactions.^66,89^ In this work, we employed light pulses of comparable duration (1 s) and of about 2 orders of magnitude lower photoexcitation density. Therefore, we expect no variation in the sign of photocapacitive current and a much lower intensity of the interfacial electric field. (ii) We developed P3HT-based planar interfaces with hippocampal neurons and demonstrated that 500 ms long, green light pulses lead to membrane hyperpolarization and silencing of the neuron spontaneous activity.^14^ Based on these two arguments, we argue that photoactivated membrane depolarization, usually observed on timescales shorter than 0.5 s and limited to a few mV, is not expected to play a significant role on experimentally observed guiding of neuronal growth.

Literature reports have also shown that intracellular calcium concentration can sizably influence the interactions with calmodulin, directly affect the activity of intercellular enzymes, and activate downstream signaling pathways, regulating the expression of proteins involved in neurite growth.^90,91^ Photoactivation of P3HT-based biointerfaces has been reported to effectively modulate the intracellular Ca^2+^ concentration in HEK-cells,^92^ PC12 cells,^16^ and human endothelial precursors.^93^ Ongoing activity, beyond the scope of the present work, is aimed at fully validating this hypothesis and to unequivocally demonstrate a direct link between polymer photoexcitation, intracellular Ca^2+^ modulation, protein expression, and downstream regulation of neuronal growth.

## 4 Conclusions

Taken together, the findings presented in this study demonstrate that 3D, light-sensitive P3HT substrates represent an effective interface for optical regulation of embryonic neuronal growth. P3HT-based microstructured substrates can be easily fabricated using already established techniques with high reproducibility and spatial resolution. Embryonic cortical neurons were successfully cultured on P3HT micropillars without affecting either the optoelectronic properties of the active material or impairing cell viability. Due to their conical shape, HAR micropillars represent a relatively soft interface that facilitates interactions with living cells and largely improves their adhesion. Neuronal somas achieve a close contact with the micropillars, mediated by membrane and cytoskeletal rearrangements. Photostimulation of primary cortical neurons on P3HT substrates resulted in a significant increase in neurite outgrowth compared to photoinert control substrates, without any deleterious effect on neuronal viability and functionality. The efficacy of optoelectrical stimulation is further enhanced by the 3D microscale topography, which induces both longer neurites and alignment along the topographically dictated angles.

The benefits offered by the proposed interface to promote neuronal growth and regeneration are manifold. First, in contrast to similar platforms employing metal electrodes, the presented device does not require an external bias, thus enabling wireless and minimally invasive regulation of cell growth. Importantly, cell responsivity to light is achieved by taking advantage of a conjugated polymer, whose long-term *in vivo* biocompatibility has already been largely documented, thus completely avoiding any genetic modification. The P3HT photoactive polymer employed in this study, as well as many other commercial and potentially suitable materials, displays optical absorption in the visible range and promotes effective neuronal growth at very low photoexcitation density, thus matching the standard technical features of light sources commonly available in any physiology or biology laboratory. Therefore, the proposed interface does not require complicated setups equipped with expensive laser systems to be practically used and implemented. Moreover, the easy processing and the excellent mechanical properties, typical of conjugated polymers, allow for employing fast, highly repeatable, and cheap fabrication techniques, like the push-coating developed here. This opens up the opportunity to quickly fabricate multiple interfaces, to parallelize this approach to different materials and cell models, as well as to rapidly develop ad hoc functionalized interfaces for specific applications. Finally, the effective coupling of topographical cues with light excitation allows for unprecedented spatial and temporal resolution, opening up the opportunity to selectively probe cell sub-populations, or even cell sub-compartments, at different phases of cell growth, in a minimally invasive manner without perturbing the incubating conditions. The use of optical excitation also carries an important and intrinsic drawback regarding future applications in deep tissues due to the scarce penetration of visible light. However, it is worth mentioning that several technological solutions, based on ultraconformable and ultrathin optical fibers, have already been successfully proposed and are currently under intensive testing for *in vivo* optogenetics applications.^94^

We believe that the presented interface holds a concrete potential as a neural engineering scaffold to promote neural growth and regeneration, to develop unprecedented strategies in regenerative medicine, and ultimately to explore technological solutions for the treatment of neurodegenerative diseases.

## Supporting Information Available

OrmoComp HAR micropillars, viability on P3HT substrates, SEM characterization of the cell-micropillar adhesion, bright field images of primary cortical neurons cultured on flat and microstructured polymer surfaces, numerical fitting values of EIS experimental data fitting, neurite growth on micropillar arrays, P3HT absorption spectrum, viability of cortical neurons after optical stimulation, average number of neurites, functionality of cortical neurons after optical stimulation of P3HT substrate, and calculation of pillar bending stiffness (PDF)
