# Supplementary material for: High Aspect Ratio and Light-Sensitive Micropillars Based on a Semiconducting Polymer Optically Regulate Neuronal Growth
Source: ACS Appl Mater Interfaces. 2021 May 13;13(20):23438–51. doi: 10.1021/acsami.1c03537 (PMC8161421; doi:10.1021/acsami.1c03537)
Supplement: Supplementary file 1 — am1c03537_si_001.pdf [file am1c03537_si_001.pdf]

SUPPORTING INFORMATION

High-Aspect-Ratio and Light-Sensitive Micropillars Based on a Semiconducting Polymer Optically Regulate Neuronal Growth

Frano Milos,[1,2,#] Gabriele Tullii,[3,#] Federico Gobbo,[3,4] Francesco Lodola,[3] Francesco Galeotti,[5] Chiara Verpelli,[6] Dirk Mayer,[1] Vanessa Maybeck,[1] Andreas Offenhäusser,[1,2,] Maria Rosa Antognazza[3,*]*

[1] Institute of Biological Information Processing IBI-3, Forschungszentrum Jülich GmbH, 52425 Jülich, Germany

[2] RWTH University Aachen, 52062 Aachen, Germany

[3] Center for Nano Science and Technology @PoliMi, Istituto Italiano di Tecnologia, 20133 Milano, Italy

[4] Physics Department, Politecnico di Milano, P.zza L. Da Vinci 32, 20133 Milano, Italy

[5] Istituto di Scienze e Tecnologie Chimiche G. Natta (SCITEC), Consiglio Nazionale delle Ricerche, 20133 Milano, Italy

[6] Istituto di Neuroscienze, Consiglio Nazionale delle Ricerche, 20133 Milano, Italy

[#] Equally contributing authors

[*] Co-corresponding authors: a.offenhaeusser@fz-juelich.de; mariarosa.antognazza@iit.it

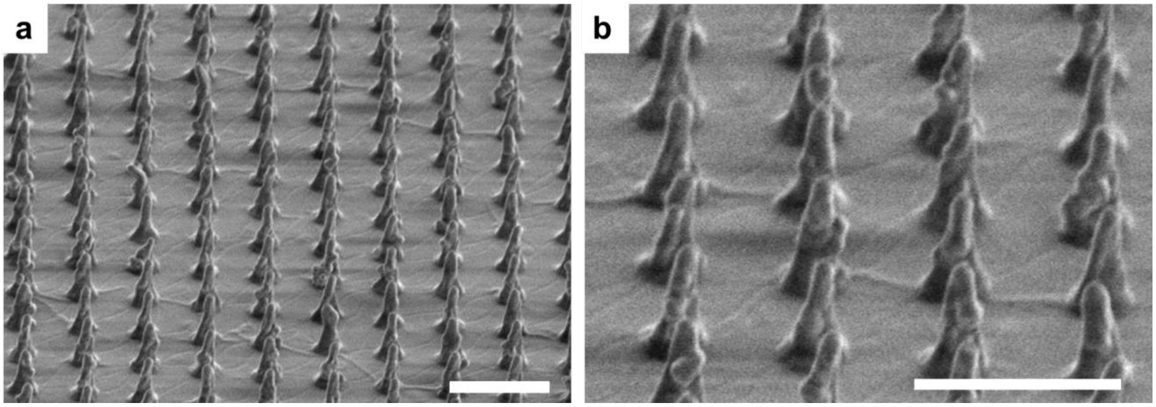

Figure S1. a) OrmoComp HAR micropillars deposited on glass substrates. **b)** A magnified image of individual OrmoComp micropillars. Scale bars: 10 µm.

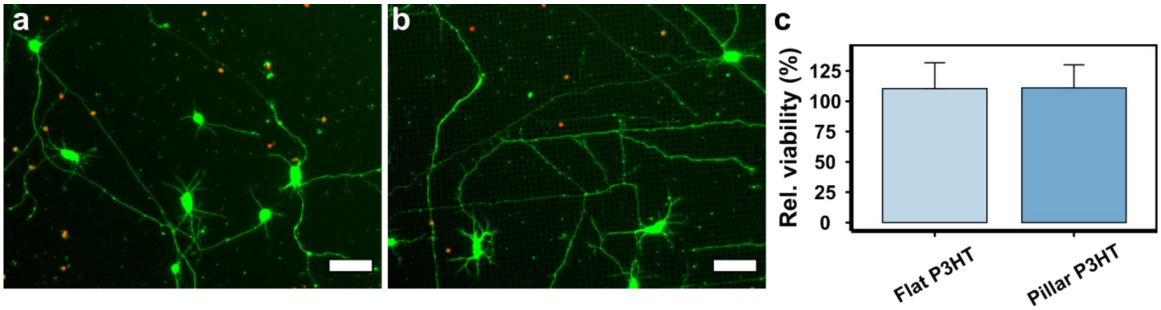

Figure S2. Neuronal viability on P3HT substrates. Cortical neurons growing on flat P3HT **(a)** and P3HT micropillars **(b)**. Cells were treated with calcein AM (viable; green) and ethidium homodimer (non-viable; red). Scale bars: 50 µm. **c)** Relative viability of cortical neurons on P3HT substrates normalized to standard glass controls after 3 DIV. Three independent experiments for each substrate were analyzed and the data presented as mean ± SE.

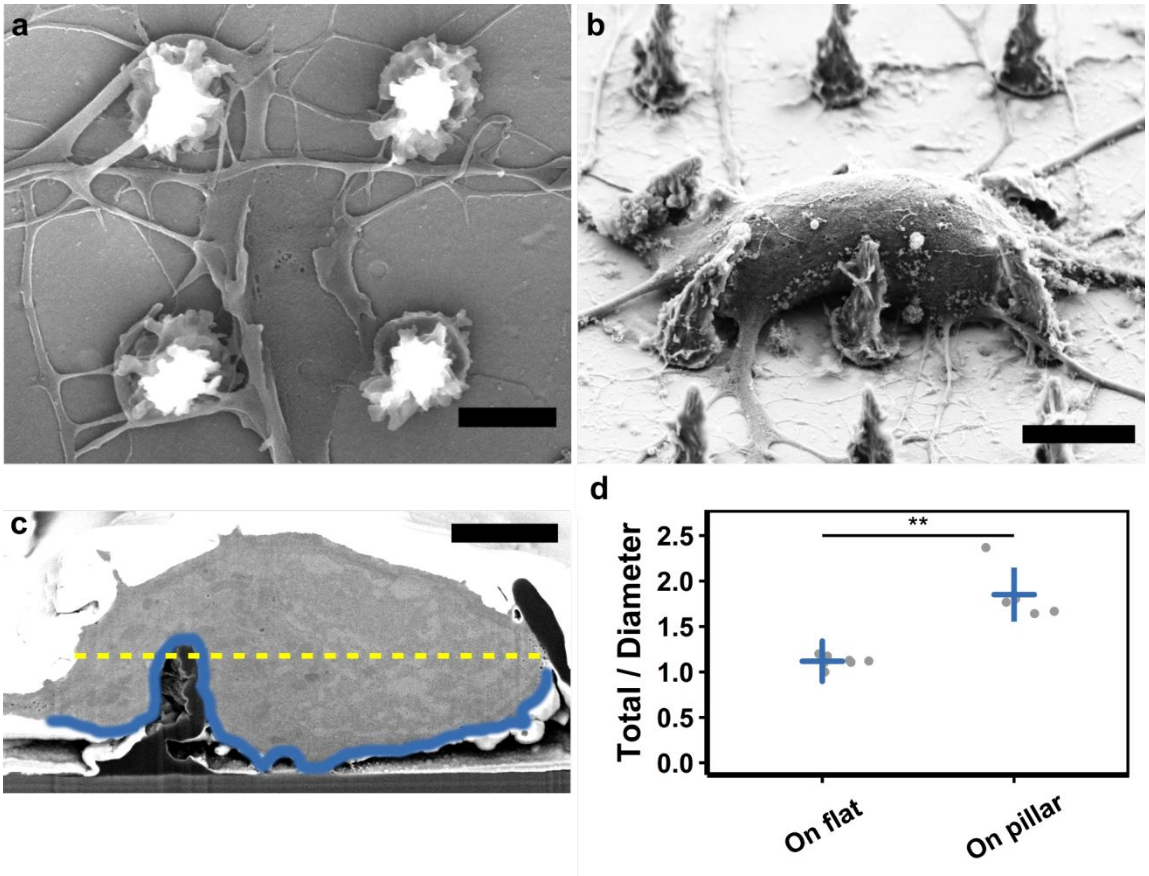

Figure S3. FIB/SEM characterization of the cell-micropillar adhesion. Representative images of neurites wrapping around the micropillars **(a)** and micropillar bending through cellular forces **(b)**. **c)** FIB cross-section of a neuronal soma positioned on the pillar. The total junctional membrane is outlined in blue while the soma diameter is denoted as a yellow dashed line. Scale bars: 2 µm for panels (a, c) and 5 µm for panel (b). **d)** Quantification of membrane wrapping around micropillars presented as the ratio of the total junctional membrane and the soma diameter. Data expressed as mean ± SD of 7 cells on flat and 5 cells on pillar and compared using the Student's t-test with Bonferroni-Holm multiple comparison correction (0.05 significance level). ** $p < 0.01$.

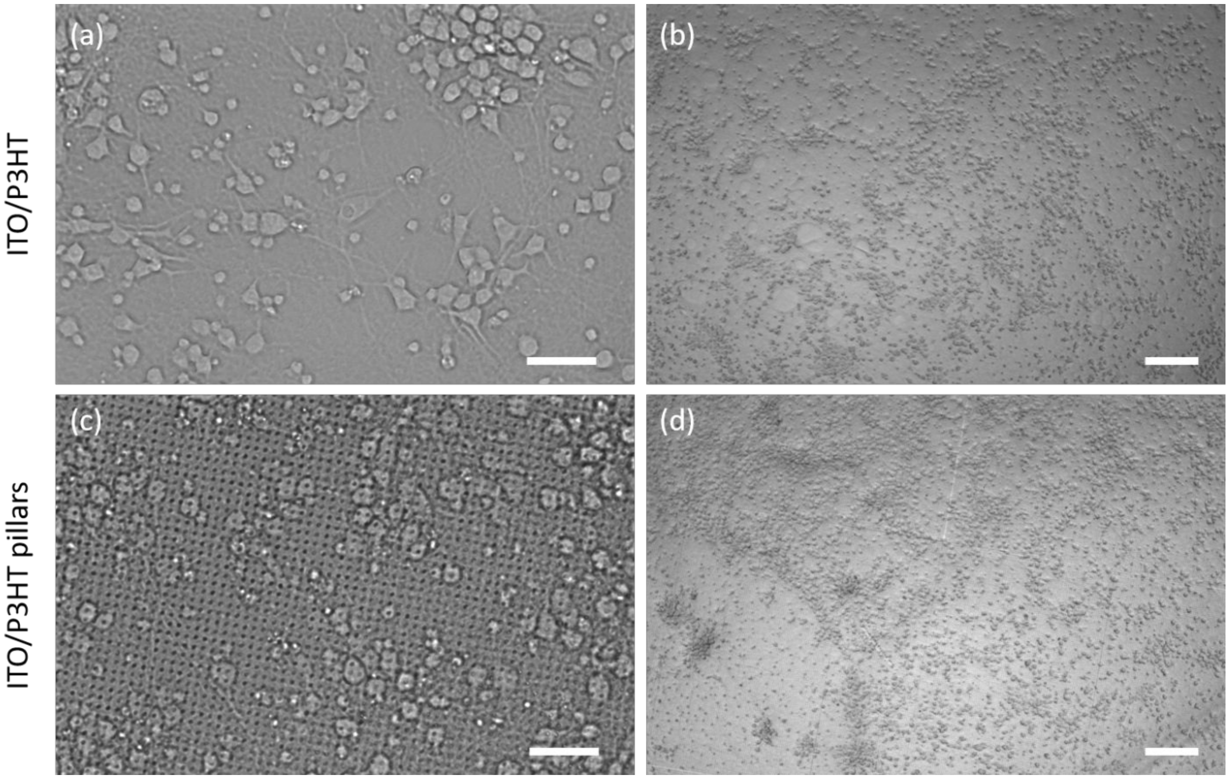

Figure S4. Bright field images of primary cortical neurons cultured on ITO/P3HT **(a, b)** and ITO/P3HT micropillars **(c, d)** substrates at decreasing magnification. Scale bars: 50 µm and 200 µm for panels (a, c) and (b, d), respectively.

Table S1. Numerical values obtained by fitting of the EIS experimental data using equivalent circuits depicted in Figure 3, panels (e-h) of the main text, normalized to the geometrical device area.

Device	R_{el} (Ω cm^2)	C_{el} (μF cm^{-2})	Q_{dl}		W_{dl}	R_{CT} (Ω cm^2)	C_p (μF cm^{-2})	R_P (Ω cm^2)	Q_c		R_{cell}	W_c
			Y_0 (μS cm^{-2})	n	Y_0 (μS cm^{-2})				Y_0 (μS cm^{-2})	n		Y_0 (μS cm^{-2})
**ITO/P3HT**	39.7	1.5 10^{-2}	4.6	0.881		1 10^7	14	4.6 10^3				
**ITO/P3HTpillars**	18.7	2.7 10^{-2}	11.2	0.896	34.3	7.4 10^4	11	29				
**ITO/P3HT+neurons**	54.8	1 10^{-2}	1.4	0.877		1.5 10^4			8.3	0.843	3.9 10^5	12.5
**ITO/P3HTpillars+neurons**	22	2.3 10^{-2}	11.9	0.960		3 10^4			7.7 10^2	0.519	2 10^4	

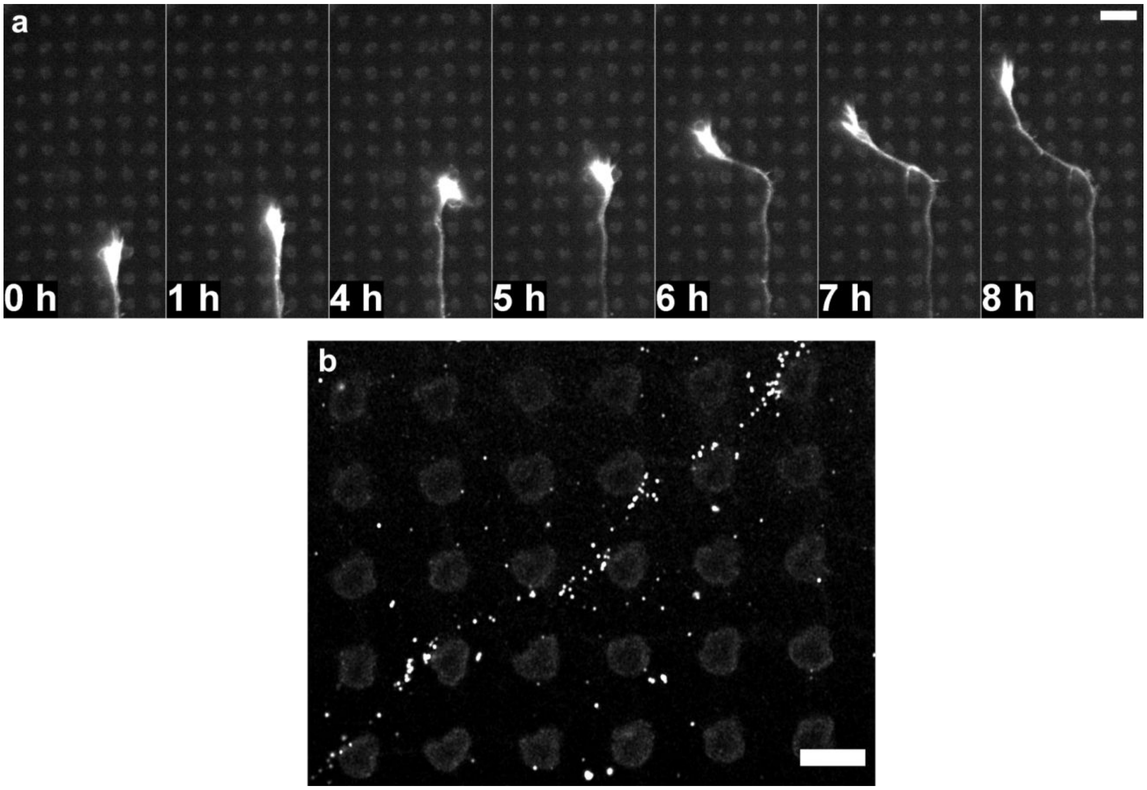

Figure S5. Neurite growth on micropillar arrays. a) Time-lapse sequence of a Lifeact-RFP-expressing growth cone extending between the pillars. Scale bar: 10 μm. **b)** Paxillin-rich adhesions (white puncta) localized on micropillars. Scale bar: 5 μm.

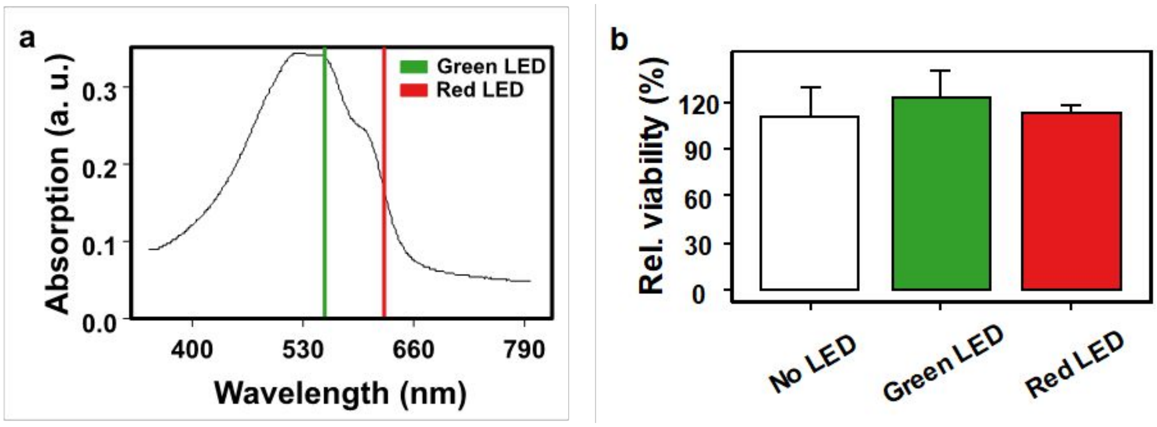

Figure S6. a) P3HT absorption spectrum. Colorized lines depict the wavelengths used for photostimulation of primary cortical neurons on P3HT substrates. **b)** Relative viability of cortical neurons after optical stimulation on P3HT substrates normalized to standard glass controls after 3 DIV. Data presented as mean ± SE of 3 independent cultures for each substrate/condition.

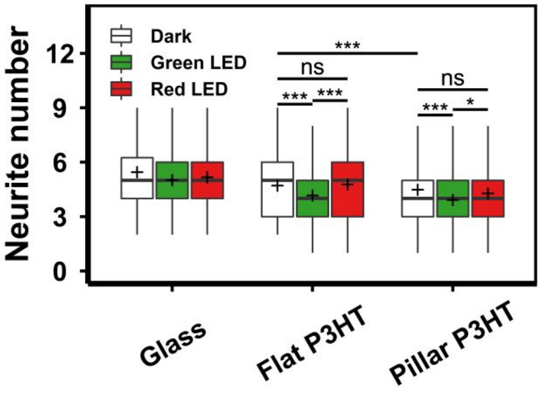

Figure S7. Average number of neurites. More than 200 neurons from 3 independent experiments were analyzed for each substrate and condition and compared using the non-parametric Mann–Whitney U test with Bonferroni-Holm multiple comparison correction (0.05 significance level). * $p < 0.05$, *** $p < 0.001$, ns – not significant.

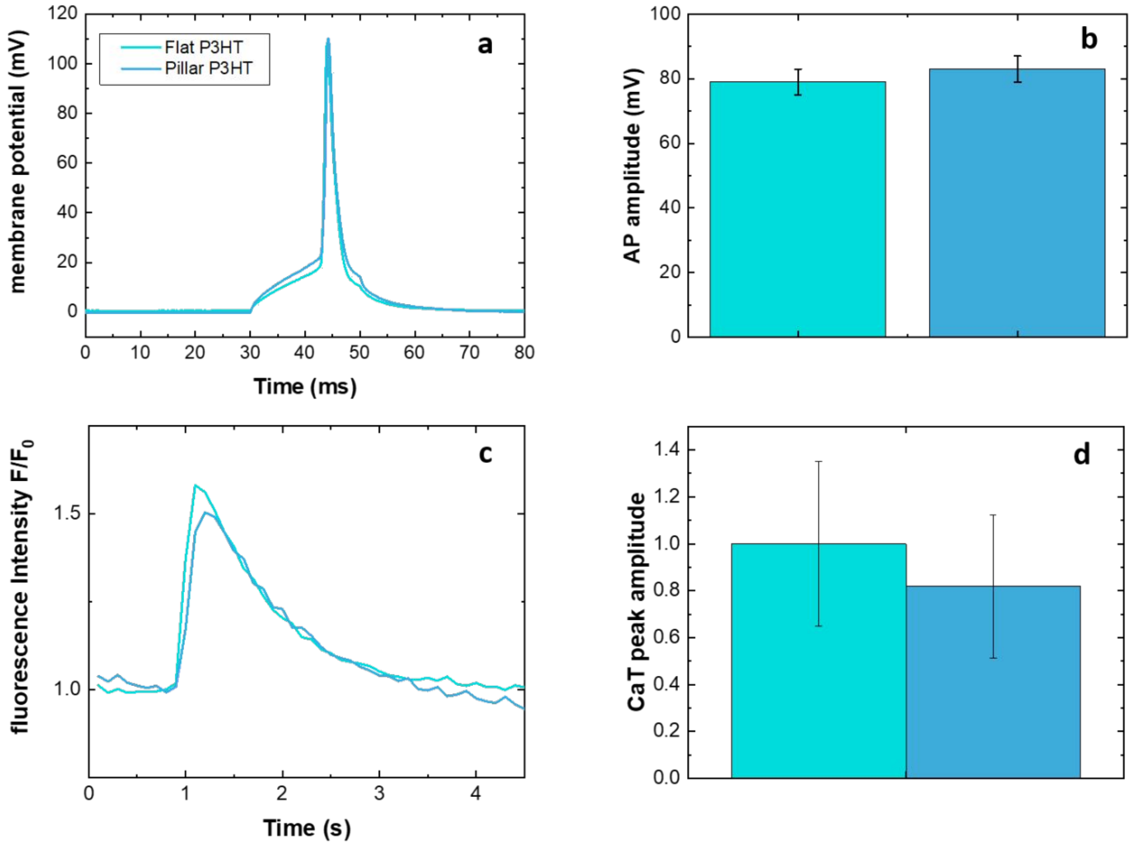

Figure S8. Patch clamp and intracellular Ca^{2+} recordings. Electrophysiology measurements were performed on cortical neurons at 14 DIV in KRH extracellular solution using a patch clamp setup based on a Axopatch 200B (Axon Instruments) in whole-cell current clamp configuration by injecting current steps of 80 pA amplitude and 20 ms time duration. **a)** Representative action potential traces of neurons grown on P3HT flat and P3HT micropillars. **b)** The average action potential peak intensity shows no significant differences between the two substrates (Student's t-test, $p > 0.05$). $n = 22$ cells for each substrate type. **c)** Representative normalized fluorescence intensity (F/F_0) traces acquired during Ca^{2+} imaging. **d)** Histograms representing the average amplitude of the first peak of the intracellular Ca^{2+} dynamics show that there is no significant difference between the pillar and planar configurations (Student's t-test, $p > 0.05$). Ca^{2+} imaging was performed using an inverted fluorescence microscope (Nikon Eclipse Ti-S), on neurons cultured on P3HT flat and pillars after 14 DIV and incubated with Fluo-4 fluorescent probe.

Calculation of pillar bending stiffness

In depth characterization of the mechanical properties of single micro/nano-structured pillars is not a straightforward task, as demonstrated in a recent work by Angeloni *et al.*[1] In fact, available techniques rely on specific instrumentation, like tensile machines, nanoindenters, or atomic force microscopy (AFM) setups that, in most of the cases, need to be coupled with scanning electron microscopes (SEM) to monitor the measurement in real-time.[2,3] Additionally, an *ad hoc* developed mathematical model is often required for reliable data analysis and interpretation. With this premise, we provide a rough estimation for the bending stiffness (K) of P3HT pillars, to critically compare its expected value with pillars reported in literature based on different materials and/or realized with different techniques.

The bending stiffness (K) of P3HT micropillars can be expressed as:

$$K = \frac{3EI}{L^3}$$

where E is the Young's modulus, I is the moment of inertia, and L is the height of the pillars.[4,5] If we assume that the cellular traction forces are exerted mainly to the upper half-part of the microstructures, we can approximate the pillars to rods and the momentum of inertia as:

$$I = \frac{\pi}{4} \cdot a^4$$

where a is the radius of the upper half-part of the pillars. Young's modulus (E) of conjugated polymers in the form of thin films reported in literature vary a lot, since they strongly depend on chemical and supramolecular properties, like regio-regularity and alignment of the polymer chains.[6,7] E values reported for P3HT lie in the range 0.1-3 GPa, in the case of thin films and nanofibers.[7–9] Based on these assumptions, we have estimated the bending stiffness (K) of the P3HT micropillars by focusing on the upper half-part characterized by an average height (L) of 3 μm and width (a) of 0.6 μm. We obtained a value of K in the range 1.1 - 33.9 μN/μm. The K values obtained for P3HT pillars are comparable to parylene C micropillars (E ≈ 4 GPa; K = 4.8-28 μN/μm)[10] and lie between the reported values for PDMS microposts (E ≈ 1 MPa; K = 0.001-1.5 μN/μm)[10,11] and inorganic materials, like silicon nanowires bundles (E ≈ 170 GPa, K = 100-1200 μN/μm).[12]

References

(1) Angeloni, L.; Ganjian, M.; Nouri-Goushki, M.; Mirzaali, M. J.; Hagen, C. W.; Zadpoor, A. A.; Fratila-Apachitei, L. E.; Ghatkesar, M. K. Mechanical Characterization of Nanopillars by Atomic Force Microscopy. *Addit. Manuf.* **2021**, *39*, 101858.

(2) Lewis, B. B.; Mound, B. A.; Srijanto, B.; Fowlkes, J. D.; Pharr, G. M.; Rack, P. D. Growth and Nanomechanical Characterization of Nanoscale 3D Architectures Grown via Focused Electron Beam Induced Deposition. *Nanoscale* **2017**, *9*, 16349–16356.

(3) Banerjee, A.; Bernoulli, D.; Zhang, H.; Yuen, M. F.; Liu, J.; Dong, J.; Ding, F.; Lu, J.; Dao, M.; Zhang, W.; Lu, Y.; Suresh, S. Ultralarge Elastic Deformation of Nanoscale Diamond. *Science (80-.).* **2018**, *360*, 300–302.

(4) Li, Z.; Song, J. H.; Mantini, G.; Lu, M.-. Y.; Fang, H.; Falconi, C.; Chen, L.-. J.; Wang, Z. L. Quantifying the Traction Force of a Single Cell by Aligned Silicon Nanowire Array. *Nano Lett.* **2009**, *9*, 3575–3580.

(5) Lin, I.-K.; Liao, Y.-M.; Liu, Y.; Ou, K.-S.; Chen, K.-S.; Zhang, X. Viscoelastic Mechanical Behavior of Soft Microcantilever-Based Force Sensors. *Appl. Phys. Lett.* **2008**, *93*, 251907.

(6) Root, S. E.; Savagatrup, S.; Printz, A. D.; Rodriquez, D.; Lipomi, D. J. Mechanical Properties of Organic Semiconductors for Stretchable, Highly Flexible, and Mechanically Robust Electronics. *Chem. Rev.* **2017**, *117*, 6467–6499.

(7) Jiang, K.; Xu, D.; Ma, Z.; Yang, P.; Song, Y.; Zhang, W. Quantifying the Mechanical Anisotropy in Poly(3-Hexylthiophene) Nanofibers. *ACS Macro Lett.* **2020**, *9*, 108–114.

(8) Jiang, K.; Wang, L.; Zhang, X.; Ma, Z.; Song, Y.; Zhang, W. Side-Chain Length Dependence of Young's Modulus and Strength in Crystalline Poly(3-Alkylthiophene) Nanofibers. *Macromolecules* **2020**, *53*, 10061–10068.

(9) Zhang, S.; Ocheje, M. U.; Luo, S.; Ehlenberg, D.; Appleby, B.; Weller, D.; Zhou, D.; Rondeau-Gagné, S.; Gu, X. Probing the Viscoelastic Property of Pseudo Free-Standing Conjugated Polymeric Thin Films. *Macromol. Rapid Commun.* **2018**, *39*, 1800092.

(10) Fohlerova, Z.; Gablech, I.; Otahal, A.; Fecko, P. SiO2-Decorated Parylene C Micropillars

Designed to Probe Cellular Force. *Adv. Mater. Interfaces* **2021**, *8*, 2001897.

(11) Sochol, R. D.; Higa, A. T.; Janairo, R. R. R.; Li, S.; Lin, L. Unidirectional Mechanical Cellular Stimuli via Micropost Array Gradients. *Soft Matter* **2011**, *7*, 4606–4609.

(12) Lin, H. I.; Kuo, S. W.; Yen, T. J.; Lee, O. K. SiNWs Biophysically Regulate the Fates of Human Mesenchymal Stem Cells. *Sci. Rep.* **2018**, *8*, 12913.
